# Production of Hydroxymethylfurfural Derivatives From Furfural Derivatives *via* Hydroxymethylation

**DOI:** 10.3389/fbioe.2022.851668

**Published:** 2022-02-15

**Authors:** Xianqing Lv, Xiaolin Luo, Xin Cheng, Jing Liu, Changzhi Li, Li Shuai

**Affiliations:** ^1^ College of Materials Engineering, Fujian Agriculture and Forestry University, Fuzhou, China; ^2^ CAS Key Laboratory of Science and Technology on Applied Catalysis, Dalian Institute of Chemical Physics, Chinese Academy of Sciences, Dalian, China

**Keywords:** furfural derivatives, hydroxymethylfurfual derivatives, hydroxymethylation, hydrocarbon fuel, hydrodeoxygenation

## Abstract

Hydroxymethylfurfural (HMF) derivatives such as 2,5-bis(hydroxymethyl)furan (BHMF) and furandicarboxylic acid (FDCA) are promising alternative of fossil-based diols and dicarboxylic acids for synthesis of polyesters such as polyethylene terephthalate (PET). However, high cost for preparing HMF from biomass discourages the commercialization of HMF-derived polyesters. Since producing furfural (FUR) from five-carbon sugars (e.g., xylose) via dehydration is an inexpensive and commercialized process, we herein reported a method to synthesize BHMF derivatives (5-(ethoxymethyl)furan-2-methanol (EMFM), 2,5-bis(hydroxymethyl)furan monoacetate (BHMFM) and 2,5-bis(hydroxymethyl)furan diacetate (BHMFD) from furfural derivatives, i.e., (2-(ethoxymethyl)furan (EMF) and furfuryl acetate (FA)). To avoid strong acid-induced side reactions (e.g., furan ring opening, condensation and carbonization), two reaction systems, i.e., a low-concentration HCl aqueous solution combined with formaldehyde and anhydrous acetic acid combined with paraformaldehyde, were found to be suitable for such a hydroxymethylation reaction and could lead to decent product yields. In order to improve the carbon utilization, condensed furanic byproducts were further converted into hydrocarbon fuels via a reported two-step hydrodeoxygenation (HDO) process. This study not only validates the possibility of synthesizing functional HMF derivatives (EMFM, BHMFM, and BHMFD) from commercially-available FUR derivatives (EMF and FA), but also provide a new way to transform condensed furanics to value-added hydrocarbon fuels.

## Introduction

**SCHEME 1:**
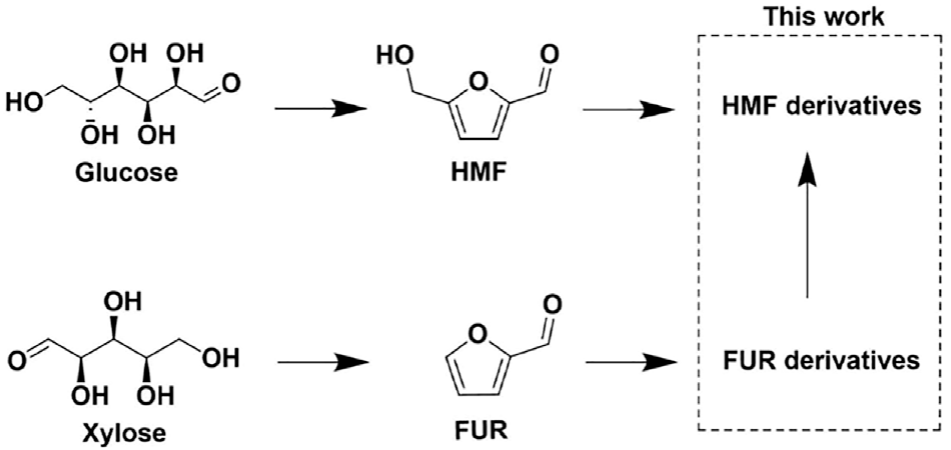
The pathway of preparing HMF and HMF derivatives from biomass.

Synthetic polymers are important material basis for promoting the development of industry and human society. Most of the commercialized polymers [e.g., polyethylene terephthalate (PET)] are synthesized based on fossil-based chemicals (e.g., terephthalic acid). However, the extensive use of fossil-based chemicals brings about severe environmental problems such as the greenhouse effect and the shortage of nonrenewable resources. Developing alternatives of fossil-based chemicals and materials from renewable biomass is a promising way to overcome or at least alleviate these problems. For example, 2, 5-furandicarboxylic acid (FDCA) can be used to substitute terephthalic acid for synthesis of polyesters such as polyethylene furandicarboxylate (PEF), which showed better performance (e.g., thermostability and elasticity modulus) than that of PET (Guan et al., 2021; Yang and Mu, 2021). Polyesters could also be synthesized by using 2,5-bis(hydroxymethyl)furan (BHMF) as an alternative of fossil-based diols (e.g., ethylene glycol). As a result, hydroxymethylfurfural (HMF) derivatives such as FDCA and BHMF have been considered as important platform chemicals for synthesizing bio-based polymers, which thereby stimulate intensive studies in preparing the two chemicals from renewable biomass (Scheme 1).

At present, FDCA is synthesized from 5-hydroxymethyl furfural (HMF) through hydrothermal (Gao et al., 2017; Cheng et al., 2021; Kandasamy et al., 2021; Yu et al., 2021) or electrochemical oxidation (Hu et al., 2021; Zhang et al., 2021). During the hydrothermal oxidation process, HMF is initially oxidized to 2, 5-diformylfuran (DFF) and/or 5-hydroxymethyl-2-furanoic acid (HMFCA), which is further converted to 5-formyl-2-furanoic acid (FFCA) and FDCA under aerobic conditions (Kandasamy et al., 2021). The reaction pathway of converting HMF to FDCA via electrochemical oxidation is similar to that of hydrothermal oxidation, but such a method showed good selectivity only at low substrate concentrations. In addition to FDCA, HMF can be also reductively converted to BHMF, which is a furanic diol for synthesis of polyesters. With the input of H_2_, different catalysts such as Ru/Co_3_O_4_ (Chen et al., 2021), Cu-Al_2_O_3_ (Rao et al., 2021), Ni-Cu/HT (Gupta et al., 2020), and CuO-Fe_3_O_4_/AC (Elsayed et al., 2020), had been developed for reducing HMF to BHMF. These studies indicate that HMF and its derivatives are important bio-based small molecules for synthesizing FDCA, BHMF and many other value-added derivatives. However, a critical issue related to HMF and its derivatives is the high cost for preparing them from biomass. HMF is generally produced via the dehydration of six-carbon carbohydrates (e.g. glucose and fructose) while the dehydration process is either inefficient due to low selectivity of HMF or costly due to energy-intensive solvent recovery and separation (Sajid et al., 2018). In contrast, dehydration of five-carbon sugars (e.g. xylose) to produce furfural (FUR) is a commercialized process and furfural has been successfully used to synthesize furan resin (Ye et al., 2021).

**FIGURE 1:**
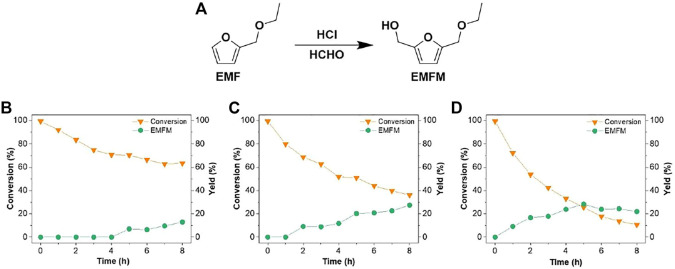
**(A)** Synthesis pathway of EMFM from EMF; time-dependent conversion of EMF and yield of EMFM at **(B)** 30°C; **(C)** 40°C; and **(D)** 50°C. Other hydroxylmethylation reaction conditions: 0.4 mmol EMF, 1 ml formaldehyde (37 wt% aqueous solution), 0.1 mmol HCl (10 µL of 36 wt% HCl aqueous solution) and 1 ml 1,4-dioxane.

**FIGURE 2:**
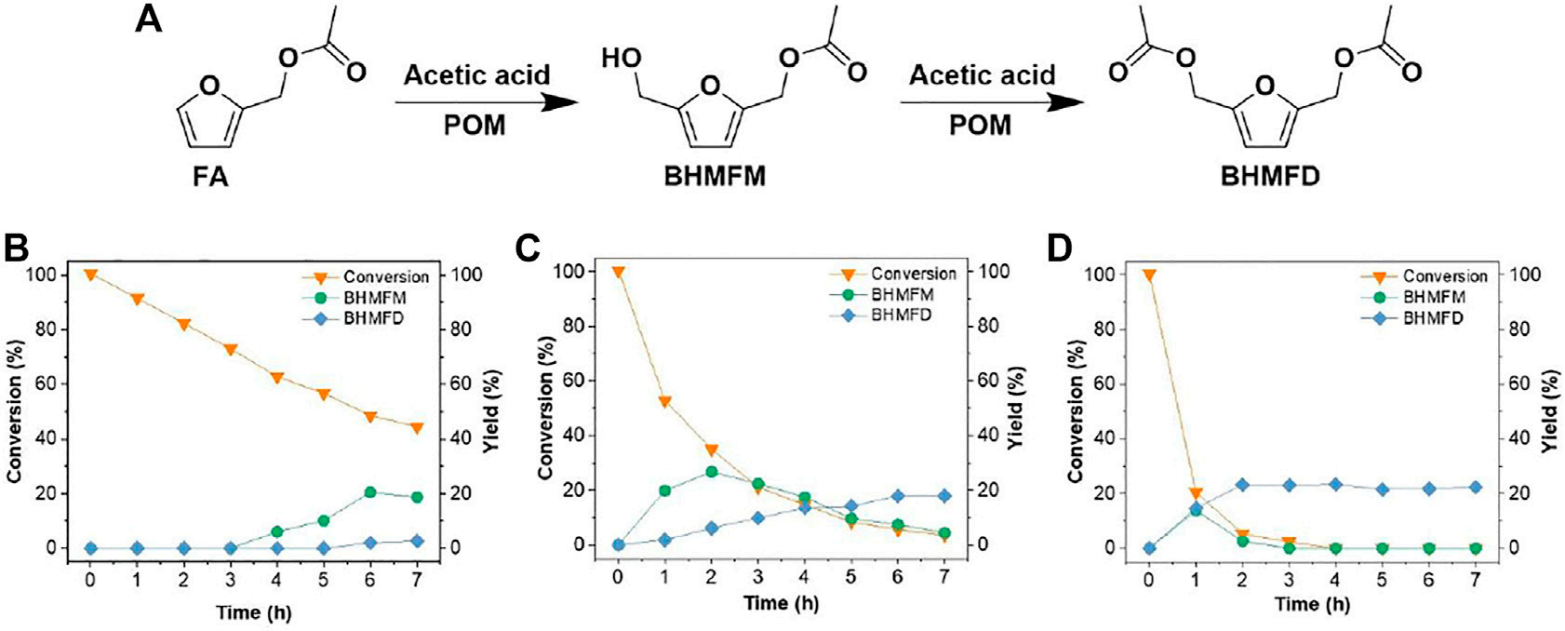
**(A)** Synthesis pathway of BHMFM and BHMFD from FA; time-dependent conversion of FA and yield of BHMFM and BHMFD at **(B)** 80°C; **(C)** 100°C; and **(D)** 120°C. Other hydroxylmethylation reaction conditions: 0.4 mmol FA, 1.7 mmol paraformaldehyde and 35.0 mmol CH_3_COOH.

As the production of FUR from five-carbon sugars such as xylose is easy and inexpensive, we believe that synthesis of HMF or HMF derivatives (e.g. BHMF ethers and esters) from inexpensive FUR or its derivatives (e.g., furfuryl acetate and 2-(ethoxymethyl)furan)) would be an interesting and potential way for further investigation. Such a pathway requires incorporation of one carbon to the C-5 position of furfural via hydroxymethylation, which was rarely reported. Therefore, in this article we proposed new reaction pathways for synthesizing BHMF derivatives (2,5-bis(hydroxymethyl)furan diacetate and 5-(ethoxymethyl)furan-2-methanol) via hydroxymethylation of furfuryl alcohol derivatives (furfuryl acetate and 2-(ethoxymethyl)furan) with formaldehyde or paraformaldehyde (Figures 1A, 2A). The reason we chose etherified or esterified furfural derivatives as starting materials was that these derivatives were more stable than FUR and furfuryl alcohol under acidic conditions. During these reactions, we found that acid could lead to the opening of furan rings, which produced carbonyl compounds that would further condense to form humins-like products. To improve the utilization efficiency of bio-based carbon resources, the oxygen-containing condensed products were converted to hydrocarbon fuels via hydrodeoxygenation. These results validate not only a new way to synthesize HMF derivatives but also a new method for utilizing condensed furan products for hydrocarbon fuels production.

## Materials and Methods

### Materials

Furfuryl acetate (99%) and 2-(ethoxymethyl)furan (98%) were purchased from Rhawn Chemical Technology Co., Ltd. (Shanghai, China). Paraformaldehyde (98%), Na_2_HPO_4_·H_2_O (98%) and NaH_2_PO_4_ (98%) were purchased from Maclin Biochemical Co., Ltd. (Shanghai, China). Pt on carbon (Pt 5 wt%), n-hexane (98%), n-hexadecane (99.5%, GC), chloroform-d (99.8%), formic acid (99%), trifluoromethanesulfonic acid (98%) and MgSO_4_ (AR) were ordered from Aladdin^®^ Biochemical Technology Co., Ltd. (Shanghai, China). Phosphotungstic acid anhydrate (99%) was purchased from Titan Scientific Co., Ltd. (Shanghai, China). Anhydrous acetic acid (99.5%), formaldehyde (37 wt%), chloroform, concentrated sulfuric acid (98 wt%) and concentrated hydrochloric acid (36 wt%) were all obtained from XiLong Scientific Co., Ltd. (Shantou, China). All reagents were used as received without further purification.

### Synthesis of 5-(ethoxymethyl)furan-2-methanol

Initially, 0.4 mmol of 2-(ethoxymethyl)furan (EMF) was mixed with 10 mmol of formaldehyde (37 wt%), 1 ml of 1,4-dioxane and 10 μL of concentrated hydrochloric acid (36 wt%) in a 15-ml vial. The vial was placed in oil bath, magnetically stirred and heated to a desired temperature (30–50 °C) for 1‒8 h. After the reaction, the vial was immediately taken out from the oil bath and cooled by tap water to room temperature. Around 0.25 mg n-hexadecane was added into the reaction liquor as an internal standard and 50 μL of the liquid was sampled and extracted by 1 ml of chloroform. The extract was dehydrated by around 200 mg anhydrous MgSO_4_. The resulting solution was analyzed by a gas chromatograph-mass spectrometer (GC-MS, SCION 436GC-SQ, Techcomp group, Shanghai, China) to identify products and the identified products were further quantitatively analyzed by a gas chromatograph (GC, SCION 436C, Techcomp group, Shanghai, China).

### Synthesis of 2,5-bis(Hydroxymethyl)Furan Monoacetate (BHMFM) and 2,5-bis(Hydroxymethyl)Furan Diacetate (BHMFD)

A mixture of furfuryl acetate (FA, 0.4 mmol), paraformaldehyde (50 mg) and anhydrous acetic acid (2 ml) was loaded into a 15-ml vial. The vial was magnetically stirred and heated at 100–120°C in an oil bath for 1‒7 h. After the reaction, the vial was immediately taken out from the oil bath and cooled by tap water to room temperature. Around 0.25 mg n-hexadecane was added into the reaction liquor as an internal standard. 50 μL of the liquid was sampled and analyzed by a GC-MS (SCION 436GC-SQ, Techcomp group, Shanghai, China) to identify products and the identified products were further quantitatively analyzed by a GC (SCION 436C, Techcomp group, Shanghai, China).

### Separation of Monomeric Furanic Products From Condensed Furanics

The solvent in the final product mixture was carefully removed with a rotary evaporator under vacuum. The monomeric furanic products in the residual mixture were extracted with 10 ml hexane three times and the residual was considered as condensed furanics and used for oil production *via* hydrodeoxygenation.

### Hydrodeoxygenation of Condensed Furanics to Hydrocarbon Fuels

The condensed furanics separated in last section was used as raw materials for production of hydrocarbon fuels (also termed as oil) through the following hydrodeoxygenation reactions.

About 300 mg of the residual product was re-dissolved with methanol (25 ml) in a 50-ml stainless steel reactor (WZD-50, Wuzhou Dingchuang Technology Co., Ltd., Beijing, China) followed by the addition of Pt/C catalyst (200 mg). The reactor was flushed three times with hydrogen gas, and then pressurized with hydrogen gas to 3 MPa. The mixture was mechanically stirred, and then heated to 250°C and kept at this temperature for 4 h. After the reaction, the reactor was cooled to room temperature and depressurized carefully. The catalyst was separated by filtration, and the reaction liquor was collected. Methanol in the reaction liquor was removed by a vacuum rotary evaporator. The residual organics was further mixed with Pt/C catalyst (200 mg), phosphotungstic acid (Li et al., 2021) (200 mg) and n-hexane (25 ml) in the reactor (WZD-50, Wuzhou Dingchuang Technology Co., Ltd., Beijing, China). The reactor was also flushed and pressurized with hydrogen gas to 3 MPa. The mixture was mechanically stirred at 300 rpm, and then heated to 250°C and reacted at the temperature for 4 h. The reactor was cooled to room temperature and depressurized carefully after the reaction. After the catalyst was separated via filtration, n-hexane in the resultant filtrate was removed by a vacuum rotary evaporator. The resultant viscous oil was used for further characterization.

### Monomeric Furanics Purification and Charicterization

#### Purification of Monomeric Furanics

Products obtained in the sections of “Synthesis of EMFM” and “Synthesis of BHMFM and BHMFD” were separated by a preparative liquid chromatograph (Sepabean machine2, Santai Technologies, Changzhou, China), which was equipped with a DAD detector and a Spherical C18 column (SW025, 20–45 μm). The following separation conditions were used: 100% phosphate buffer (pH 6.5, 10 mM) at 5 ml/min for 2 min, increasing volumetric ratio of acetonitrile from 20 to 100% with 35 min and keeping flow rate of eluent at 5 ml/min, 100% acetonitrile at 5 ml/min for 10 min (McKenna et al., 2015). UV wavelengths at 225 nm for EMFM and at 230 nm for BHMFM and BHMFD collections were selected, respectively. Once a UV signal was detected, the fraction containing the targeted products was automatically collected. The structure of the collected fraction was identified by GC-MS, and then further analyzed by NMR.

#### Identification of Monomeric Furanics

Products separated by the preparative liquid chromatograph were initially identified by a GC-MS (SCION 436GC-SQ, Techcomp group, Shanghai, China) that was installed with a SCION-5 MS column (30 m × 0.32 mm × 0.25 μm). The following temperature program was used for GC-MS analysis: the column temperature was initially set at 50°C and held at the temperature for 5 min, heated at a rate of 10°C/min to 300°C and held at 300°C for 5 min.

After the solvent was removed from the fraction collected from the preparative liquid chromatograph, about 5–10 mg of the purified product was dissolved in 0.5 ml chloroform-d for nuclear magnetic resonance (NMR) spectrum analysis. All ^1^H-NMR spectra were acquired on a Bruker Ascend™ 600 NMR Spectrum with an operating frequency of 600 MHz.

#### Quantitation of Monomeric Furanics

The products in the prepared samples (Sections of “Synthesis of EMFM” and “Synthesis of BHMFM and BHMFD”) were quantitatively analyzed by GC (SCION 436C, Techcomp group, Shanghai, China). The following temperature program was used for GC analysis: the column temperature was initially set at 50°C and held at 50°C for 5 min, then heated at a rate of 10°C/min to 300°C and held at 300°C for 5 min.

The concentrations of reactants and products in chloroform solutions were quantified by a well-known effective carbon number method (Shuai et al., 2016). The conversion of reactants and the yield of products were thereby calculated as follows:

$$\mathrm{Reactant}\;\mathrm{conversion}\;\left(\text{\%}\right)\frac{mol\;of\;\;reacted\;reactant}{Initial\;mol\;ofreactant}\times 100$$

$$\mathrm{Product}\;\mathrm{yield}\;\left(\text{\%}\right)\frac{mol\;of\;product}{Innitial\;mol\;of\;reactant}\times 100$$

### Elemental Analysis of Condensed Furanics Before and After Hydrodeoxygenation

To evaluate the efficiency of hydrodeoxygenation reactions and the quality of hydrocarbon fuels, the contents (wt%) of C and H elements in the prepared oil samples were analyzed by an element analyzer (Vario EL Cube, Elementar, Germany). Prior to the elemental analysis, solvent-free viscous oils obtained in the Section of “Hydrodeoxygenation of condensed furanics to hydrocarbon fuels” were further dried in a vacuum drying oven at 80°C for 48 h and then ground to powder in an agate mortar. The contents of O element in the samples was calculated by assuming that the total content of C, H and O elements for each sample was 100%.

## Results and Discussion

This study intends to introduce a hydroxymethyl group (-CH_2_OH) at the C-5 position of furan derivatives (2-(ethoxymethyl)furan (EMF) and furfuryl acetate (FA)) via electrophilic addition of formaldehyde for synthesizing 2,5-bis(hydroxymethyl)furan (BHMF) derivatives (5-(ethoxymethyl)furan-2-methanol (EMFM), 2,5-bis(hydroxymethyl)furan monoacetate (BHMFM) and 2,5-bis(hydroxymethyl)furan diacetate (BHMFD)) (Figure 1A, Figure 2A). Because furanics are unstable under acidic conditions, the effects of different acids such as hydrochloric acid, sulfuric acid, formic acid, acetic acid and trifluoromethanesulfonic acid on the hydroxymethylations of EMF and FA were initially investigated.

### Synthesis of EMFM From EMF

The results show that the strength of the acid in the reaction system has a great influence on the reaction. When the acidity used in the reaction medium was too high, the substrate was easily carbonized due to the acid-catalyzed ring opening of furan rings and severe condensation of the ring-opening products such as aldehydes or ketones. As such, strong acidity-induced carbonization reactions (e.g., entries 7,8,10, and 11 in Supplementary Table S1; entries 3,4,6,8,9, and 10 in Supplementary Table S2) resulted in dark colored reaction liquors and no hydroxymethylated product was detected in these reactions (Supplementary Table S1, Supplementary Table S2). Low-concentration hydrochloric or and anhydrous acetic acid exhibited better balance between acid-catalyzed hydroxymethylation and carbonization reactions, resulting in decent product yields. Therefore, these two catalytic systems were further studied for hydroxymethylation of furfuryl alcohol derivatives.

The results above demonstrated that low-concentration HCl aqueous solution was more beneficial than other reaction systems to facilitate the hydroxylmethylation reaction of EMF with formaldehyde. Since the acidity would be an important factor affecting the hydroxylmethylation reaction efficiency, the effect of HCl aqueous solution loading was thereby further investigated. 5-(ethoxymethyl)furan-2-methanol (EMFM), a hydroxylmethylated product of EMF, was identified by MS (Supplementary Figure S1) and ^1^H-NMR (Supplementary Figure S2) and quantified by GC (Supplementary Figure S3), when the reaction between EMF (0.4 mmol) and formaldehyde (1 ml of 37 wt% aqueous solution, 10 mmol) was catalyzed by 10 µL of 36 wt% HCl aqueous solution (0.1 mmol H^+^) in 1 ml of 1,4-dioxane at 50°C for 7 h. However, without the change of other reaction conditions, increasing the loading of 36 wt% HCl aqueous solution to 20 µL (0.2 mmol H^+^) resulted in a dark reaction solution and reduced hydroxylmethylated product. This could be mainly caused by the increased concentration of H^+^ in water and 1, 4-dioxane mixture, which could accelerate the ring opening reactions of furanics (EMF and/or EMFM) and the condensation (e.g., aldol condensation) of corresponding ring-opening products (Nakagawa and Tomishige, 2012). Meanwhile, we did not observe any hydroxylmethylated product with a decreased acid loading of 1 µL (0.01 mmol H^+^) and a reaction time of 4 h at 60°C, further indicating that acid-catalyzed ring opening and condensation reactions was kinetically faster than the hydroxylmethylation reaction of EMF With an appropriate loading (10 μL, 0.1 mmol H^+^) of HCl aqueous solution, the effects of reaction temperature and time on the hydroxylmethylation efficiency of EMF were further investigated. Overall, increasing the reaction temperature and time facilitated the EMF conversion and the formation of hydroxylmethylated product (EMFM). When the reaction temperature was 30°C, no desired product was detected in the early stage of the reaction (<4 h) (Figure 1B); when the reaction temperature was increased to 40°C, the EMFM yield gradually increased with the increase of the reaction time (Figure 1C). At an elevated reaction temperature of 50°C, the maximum yield (28.3 mol%) of EMFM was achieved at 5 h (Figure 1D) but the EMFM yield slightly decreased at an extended reaction time (6‒8 h) likely due to the condensation of furanics. With the same loading (10 μL, 0.1 mmol H^+^) of HCl aqueous solution, severe condensation or carbonization was observed when a higher temperature of 60°C was used. High-molecular-weight products (300–1,000 Da) (Entry 1 in Supplementary Table S1) observed in the GPC results (Supplementary Figure S9) confirmed the occurrence of condensation during the reaction. In the reaction system of HCl and formaldehyde aqueous solution, these results indicated that a high reaction temperature could significantly increase the rate of side reactions (furan ring opening and condensation), resulting in a serious decrease in the yield of the hydroxylmethylated product.

### Synthesis of BHMFM and BHMFD From FA

Under the similar conditions, above results promoted us to conduct the hydroxymethylation reaction of FA (entry 1 in Supplementary Table S1) with formaldehyde and HCl aqueous solutions in 1,4-dioxane at 50 °C for 1 and 5 h. However, no hydroxymethylated products were detected after replacing the EMF with FA (entry 11 in Supplementary Table S2). In addition to the furan ring opening (Liang et al., 2017) and aldol condensation of ring opening products, we infer that in the presence of water, acid could also catalyze the hydrolysis of the ester linkage of FA to form furfuryl alcohol, which could easily condense. Such side reactions could occur for the newly formed hydroxylmethylated products of FA. The hydroxyl groups could be protonated to form a carbonium ion, which could attack the electron-rich furan ring to form condensed products (He et al., 2021).

To avoid the hydrolysis of the ester linkages of FA and condensation reactions caused by strong acid in the aqueous solution, we therefore explored the hydroxymethylation reaction of FA using a weak anhydrous acid (i.e., anhydrous acetic acid) and paraformaldehyde (Entry 1 in Supplementary Table S2) instead of formaldehyde and HCl aqueous solutions. In an anhydrous reaction medium, a hydroxymethylated product of FA (2,5-bis(hydroxymethyl)furan monoacetate (BHMFM)) was identified by MS (Supplementary Figure S4) and ^1^H-NMR (Supplementary Figure S5) under an elevated temperature from 80 to 120°C. With acetic acid in the reaction system, BHMFM was further esterified by acetic acid to produce another derivative, 2,5-bis(hydroxymethyl)furan diacetate (BHMFD), which was also confirmed by MS (Supplementary Figure S6) and ^1^H-NMR (Supplementary Figure S7). The results indicated that anhydrous acidic environment was favorable to the hydroxymethylation of FA while the aqueous reaction system (e.g., formaldehyde and HCl aqueous solution) was indeed not favorable to the hydroxylmethylation of FA under acidic conditions. At a mild reaction temperature of 80°C, no BHMFM was detected within 3 h. After that, BHMFM yield gradually increased with the extension of the reaction time (Figure 2B). When the reaction temperature was elevated to 100 °C, BHMFM was quickly formed within 2 h. Further increasing the reaction time resulted in decreased BHMFM yields (Figure 2C). This was mainly caused by the esterification of BHMFM to form BHMFD (Figure 2A). The acetylation of BHMFM to form BHMFD was more evident at 120°C than those at 100°C (Figure 2D). At a reaction time of 3 h at 120°C, BHMFM was almost completely esterified to form BHMFD while the total yield of BHMFM and BHMFD was not improved at elevated reaction temperatures (>120 °C).

*In-situ* esterification could be considered as a protection strategy to prevent the condensation of BHMFM in acidic reaction system. However, the yield of BHMFM or BHMFD was not beyond 30 mol% under the investigated conditions. Similar to the hydroxymethylation reaction of EMF, this would be mainly caused by different reaction rates of target (hydroxymethylation and esterification) and side reactions (dehydration, furan ring opening and condensation). First, it is difficult to improve the yields of BHMFM and BHMFD if the ring-opening rate of furan rings is faster than the hydroxymethylation rate of FA. Second, even the reaction rate of hydroxymethylation is faster than that of furan ring opening, newly formed hydroxymethyl group could be immediately protonated by hydrogen proton and dehydrated to produce carbonium ions in an acidic medium. Carbonium ions are active components that would initiate methylene-bridging condensation products, making the esterification of BHMFM difficult and also resulting in low yields of BHMFM and BHMFD.

### Synthesis of Hydrocarbon Fuels From Condensed Furanic Products

From the perspective of kinetics, it can be seen that no matter how to optimize the reaction conditions, part of the furanics would condense to form higher-molecular-weight products (Supplementary Figure S9). Such an issue is also associated with many other studies involving HMF, furfural and their derivatives (Randolph et al., 2018; Chang et al., 2020; Modugno and Titirici, 2021). In addition to the synthesis of hydroxylmethylated products, we tentatively hydrogenated the condensed furanic products into hydrocarbon fuels in order to improve carbon utilization. Oligomerization of furanics followed by hydrodeoxygenation (HDO) reactions to produce hydrocabron fuels and lubricants have been intensively studied (Li et al., 2021). Herein, we investigated the possibility of hydrogenating the condensed furanics into hydrocarbon fuels.

**FIGURE 3:**
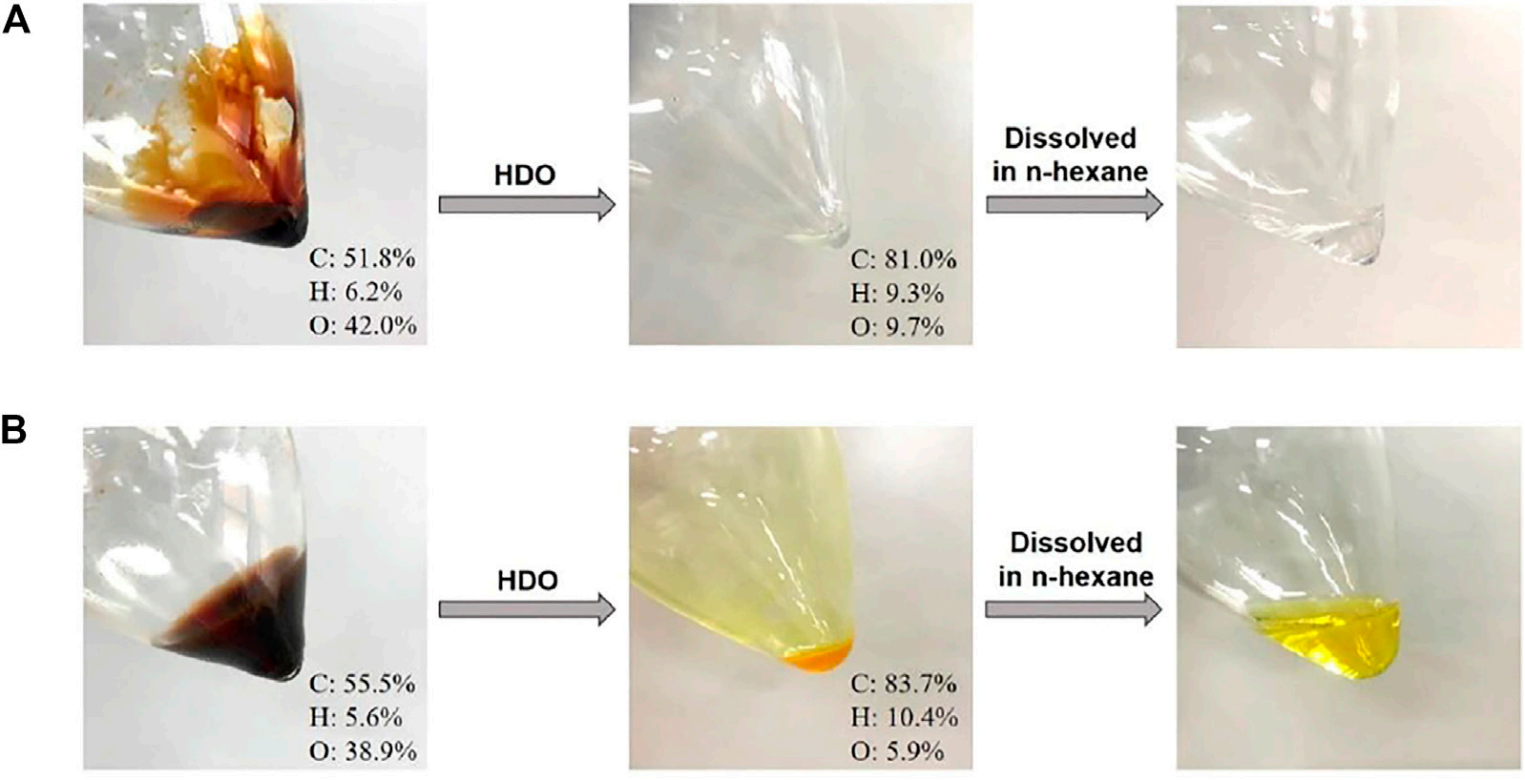
Images of condensed furanic products before and after HDO reactions. **(A)** The condensed mixture resulting from FA reacting with paraformaldehyde in CH_3_COOH; **(B)** The condensed mixture resulting from EMF reacting with formaldehyde in 1,4-dioxane.

The residue after the removal of furanic monomers via hexane extraction was considered as condensed mixture and used to produce hydrocarbon fuels *via* hydrodeoxygenation. According to a previously reported procedure (Huber et al., 2005), we used two-step HDO reactions to remove oxygen-containing functional groups from the condensed furanics. The first step was to partially hydrogenate the oxygenated furanic products to increase its solubility in the hydrophobic solvents that was used in the second step; the second step was to near completely remove oxygen from the oxygenated furanic products to obtain hydrocarbon fuels. After these HDO reactions, the color of EMF- and FA-derived oily products changed from dark to colorless and light yellow (Figure 3), respectively. Moreover, the oily products produced by HDO reactions could be well dissolved in n-hexane to form homogeneous and transparent solutions (Figure 3), indicating that these condensed furanics were successfully hydrodeoxygenated and the resultant oily products can be possibly used as drop-in hydrocarbon fuels or additives in gasoline or diesel.

The element contents of the condensed furanics before and after HDO reactions were further analyzed to confirm the occurrence of hydrodeoxygenation. It can be seen from Figure 3 that, the oxygen content of EMF-derived oily products was significantly decreased from 38.9 to 5.9% after HDO reactions, at which the contents of C and H in HDO products were increased from initial 55.5 and 5.6%–83.7 and 10.4% (Supplementary Table S3), respectively. Similar results were also obtained for that of FA-derived oily products (Supplementary Table S3), where O content decreased from 42.0 to 9.7% and C and H contents increased from 51.8 to 6.2%–81.0 and 9.3%, respectively. These results indicate that oxygen was efficiently removed from EMF- and FA-derived condensates to form hydrophobic hydrocarbon fuels with good solubility in n-hexane. As it was known, HMF-involved reactions generally lead to many condensed products, this preliminary study opened up a new way to produce hydrocarbon fuels from condensed furanics products which has been considered as waste, improving the carbon utilization efficiency.

## Conclusion

In this study, HMF derivatives, i.e., EMFM, BHMFM and BHMFD were synthesized using FA and EMF as raw materials through acid-catalyzed hydroxymethylation, proving the possibility of making six-carbon HMF derivatives from five-carbon furfural derivatives. Although such pathway is feasible, achieving high yields of hydroxymethylated products in these reactions seems challenging due to the paralleled ring-opening reaction of furanics and condensation of the resulting ring-opening products. Therefore, future research would be development of more efficient catalysts or reaction systems to suppress these side reactions to improve the selectivity of the targeted products. Alternatively, the by-products of these reactions were successfully converted into oil through hydrodeoxygenation reactions in order to improve the carbon utilization of furanics. This study provides a new route to synthesize the intermediate products of BHMF and FDCA from easily available raw materials and also prove the feasibility of converting condensed furanic by-products into drop-in hydrocarbon fuels.

## Data Availability

The original contributions presented in the study are included in the article/Supplementary Material, further inquiries can be directed to the corresponding authors.
