# Supplementary material for: Production of Hydroxymethylfurfural Derivatives From Furfural Derivatives via Hydroxymethylation
Source: Front Bioeng Biotechnol. 2022 Feb 15;10:851668. doi: 10.3389/fbioe.2022.851668 (PMC8886139; doi:10.3389/fbioe.2022.851668)
Supplement: Supplementary file 1 [file DataSheet1.docx]

**Supplementary Material for**

Production of hydroxymethylfurfural derivatives from furfural derivatives via hydroxymethylation

**Xianqing Lv^†, 1^, Xiaolin Luo^†, 1^, Xin Chen^1^, Jing Liu^1^, Changzhi Li^2,^ *, Li Shuai^1,^ ***

^1. College of Materials Engineering, Fujian Agriculture and Forestry University, Fuzhou 350002, China^

^2. CAS Key Laboratory of Science and Technology on Applied Catalysis, Dalian Institute of Chemical Physics, Chinese Academy of Sciences, Dalian, 116023 China^

^†^These two authors have contributed equally to this work.

* Correspondence:

Li Shuai

[lishuai@fafu.edu.cn](mailto:xluo53@163.com)

Changzhi Li

licz@dicp.ac.cn

**This PDF file includes：**

**Figure S1. Mass spectrum of EMFM.**

**Figure S2****. NMR spectrum of EMFM.**

**Figure S3. Gas chromatograms of EMFM.**

**Figure S4. Mass spectrum of BHMFM.**

**Figure S5. NMR spectrum of BHMFM.**

**Figure S6. Mass spectrum of BHMFD.**

**Figure S7. NMR spectrum of BHMFD.**

**Figure S8. Gas chromatograms of BHMFM and BHMFD.**

**Figure S9. GPC of sample that 0.4 mmol EMF, 1 mL formaldehyde (37 wt% aqueous solution), 0.1 mmol HCl (10 µL of 36 wt% HCl aqueous solution), and 1 mL 1,4-dioxane were reacted at 50°C for 1h, 5h and 7h.**

**Table S1. Effects of acid and aldehyde on the hydroxymethylation of EMF.**

**Table S2. Effects of acid and aldehyde on the hydroxymethylations of FA .**

**Table S3. Elemental analysis results.**

**
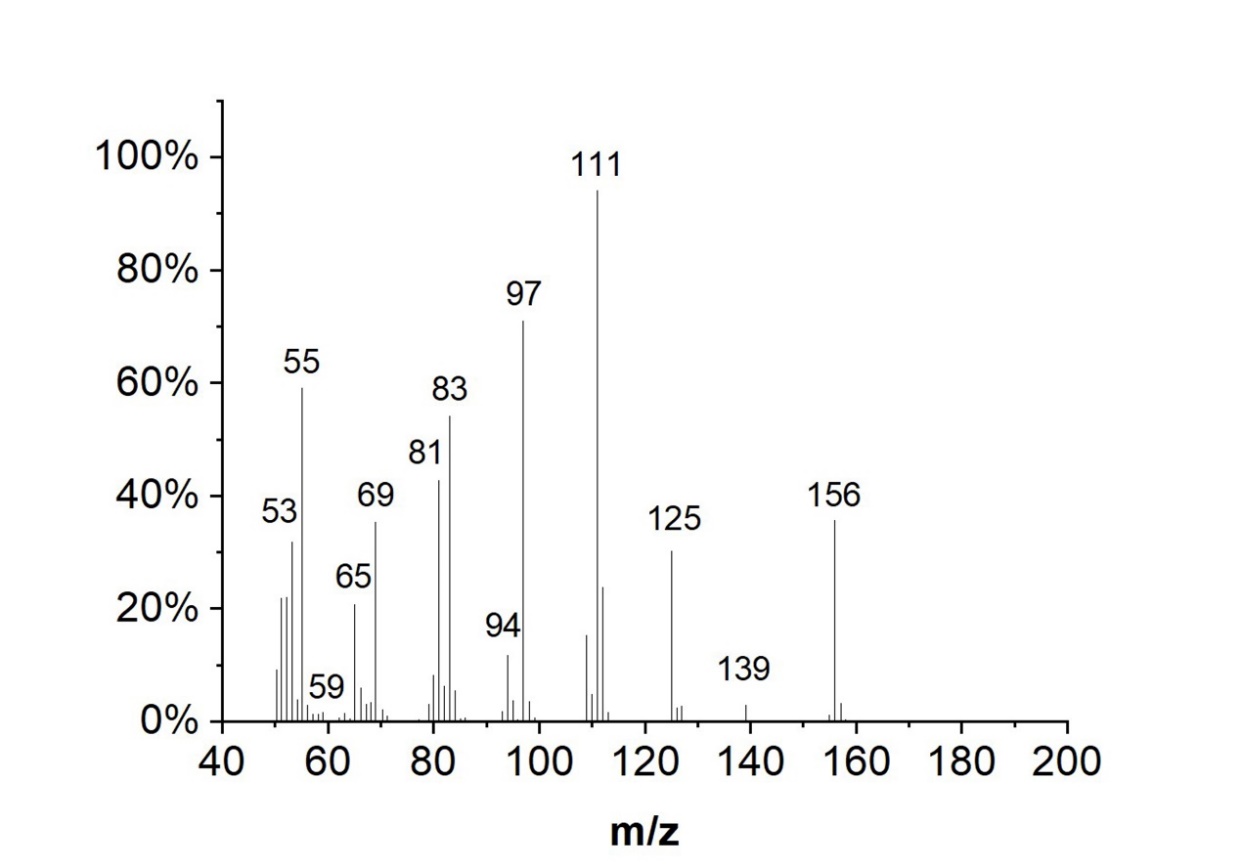
**

Figure S1. Mass spectrum of EMFM.


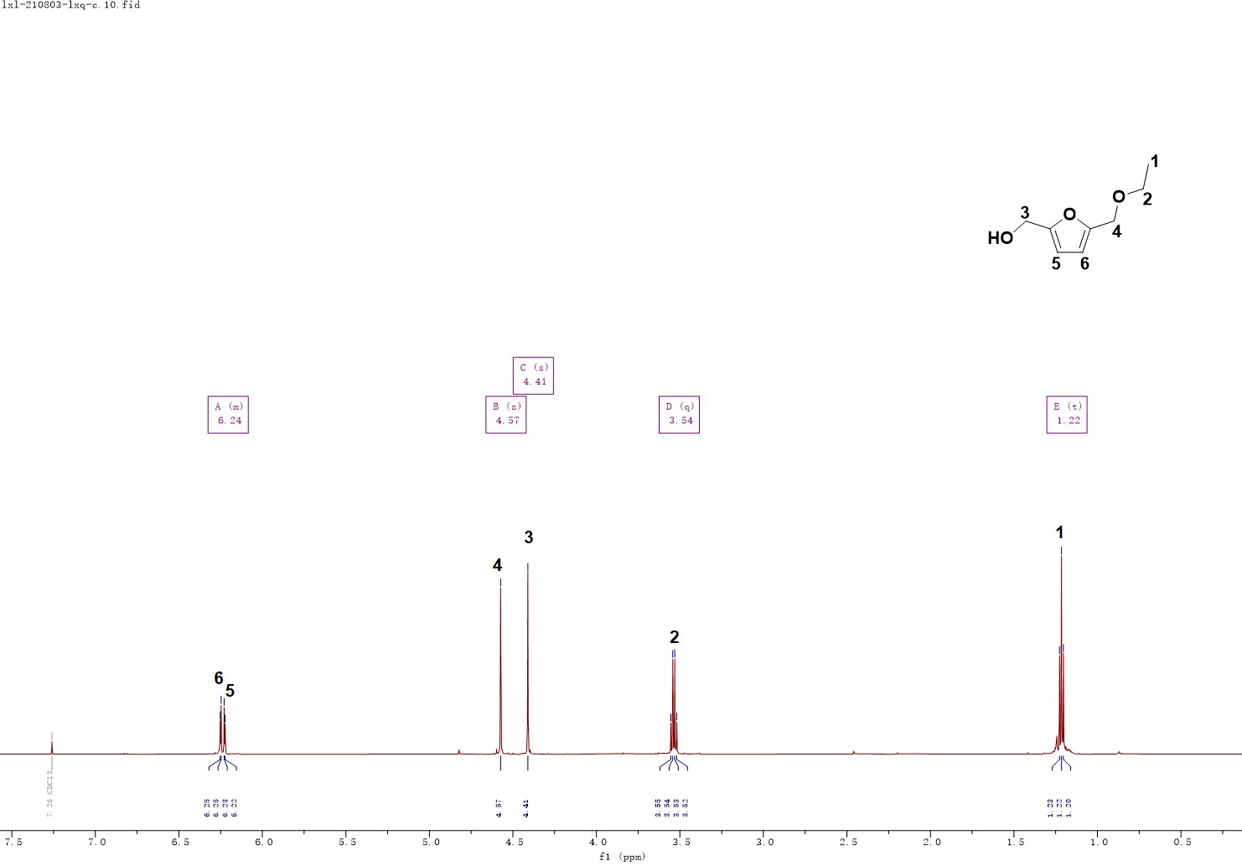


Figure S2. NMR spectrum of EMFM.


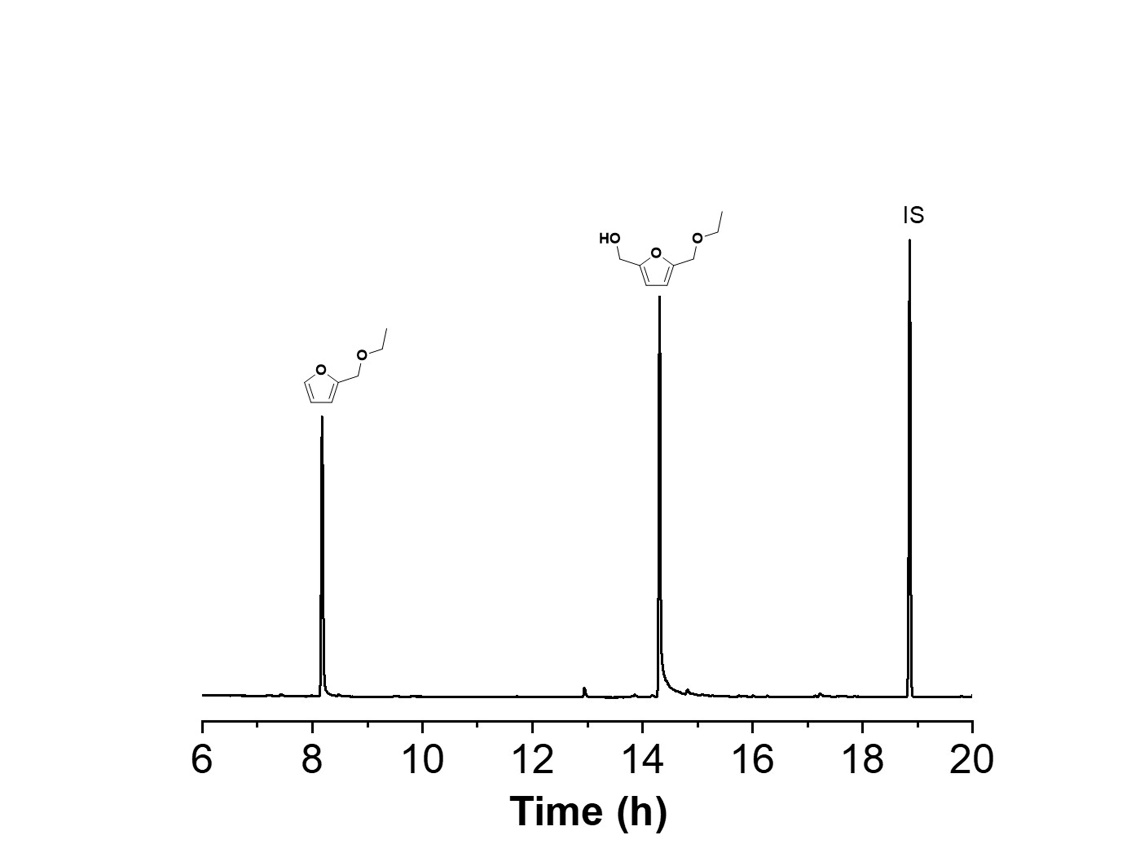


Figure S3. Gas chromatograms of EMFM. Hydroxylmethylation reaction conditions: 0.4 mmol EMF, 1 mL formaldehyde (37 wt% aqueous solution), 0.1 mmol HCl (10 µL of 36 wt% HCl aqueous solution), and 1 mL 1,4-dioxane were reacted at 50^ο^C for 5h. n-hexadecane was used as an internal standard (IS).

**
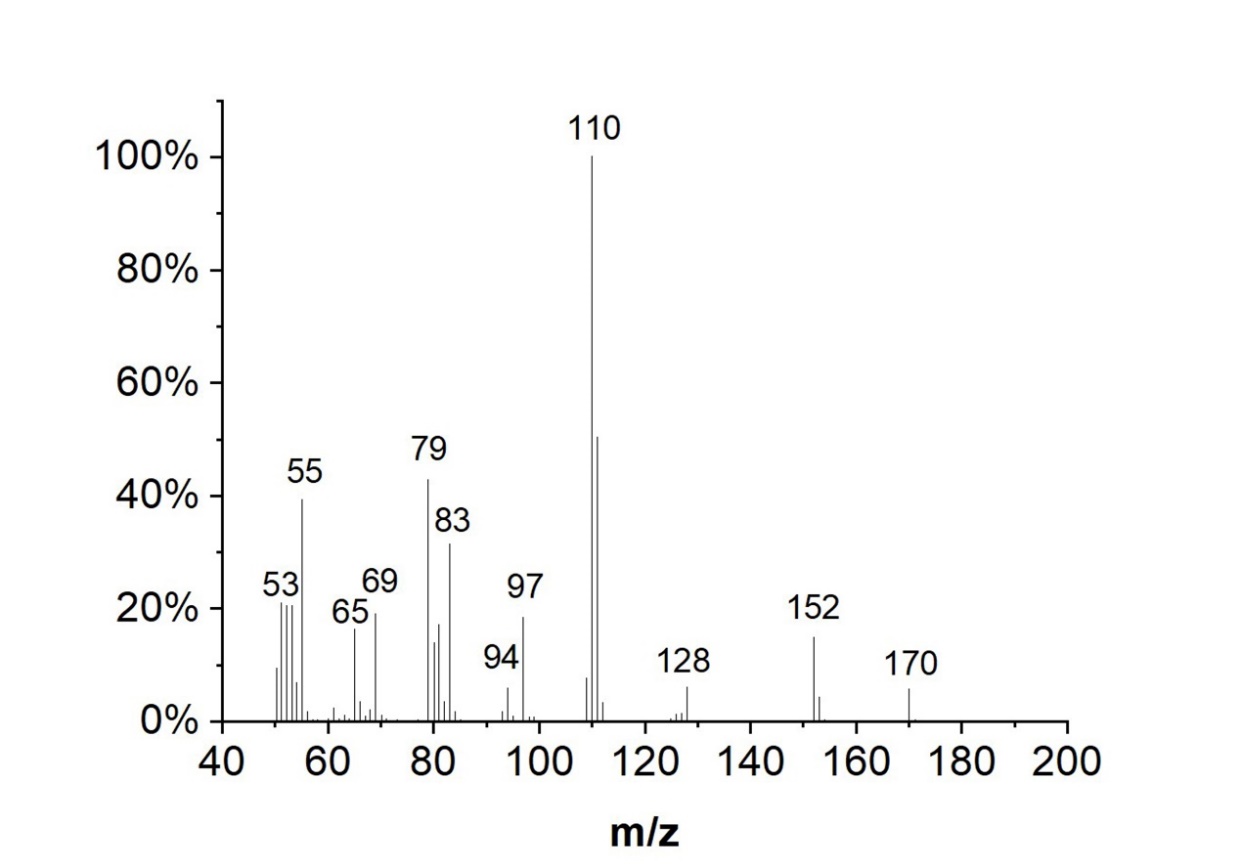
**

Figure S4. Mass spectrum of BHMFM.

**
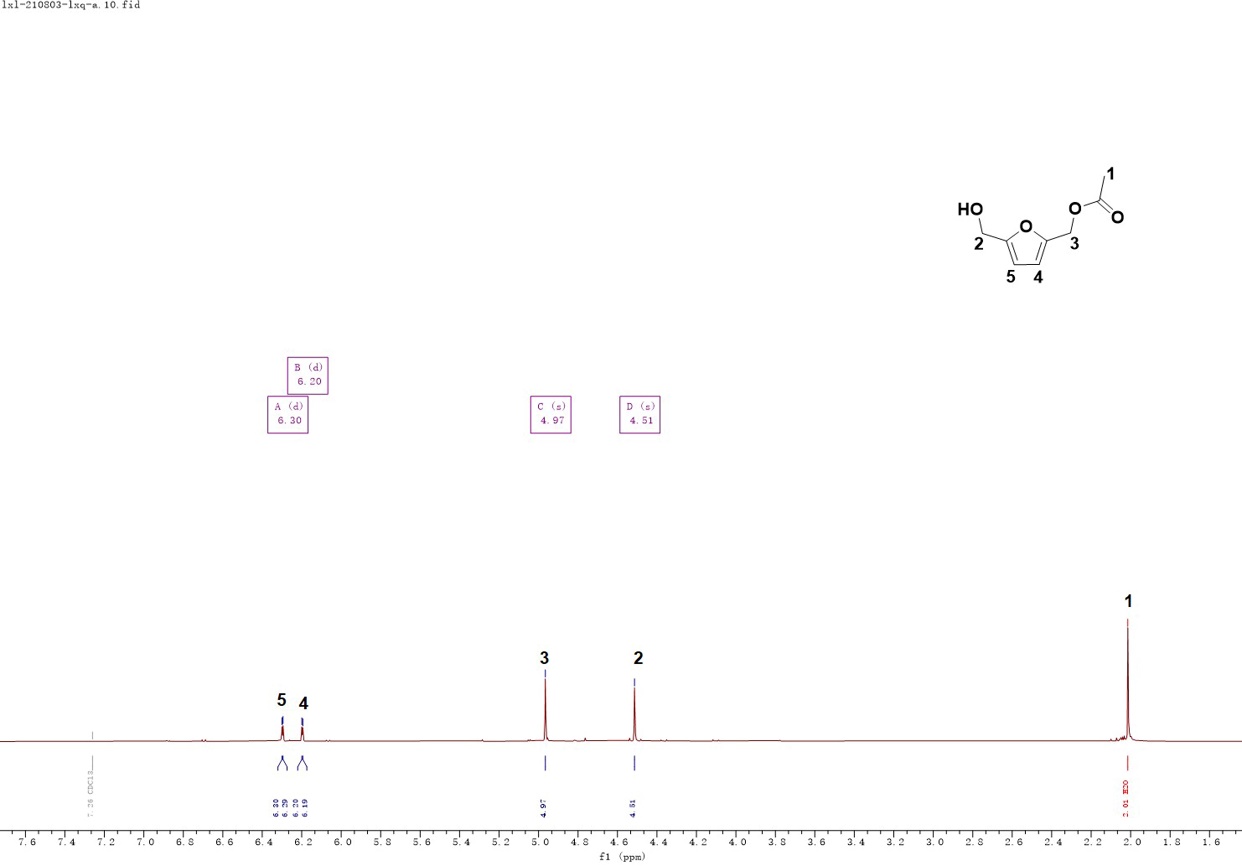
**

Figure S5. NMR spectrum of BHMFM.

**
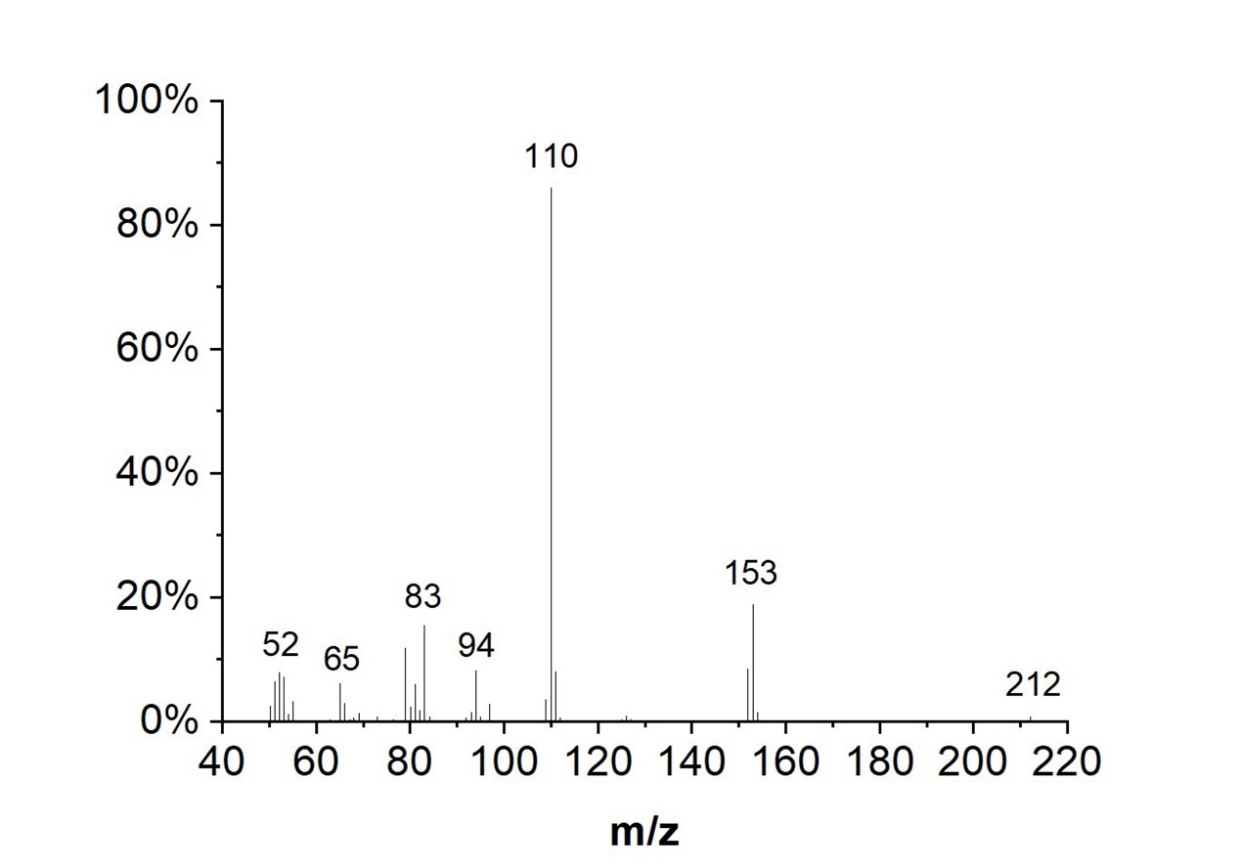
**

Figure S6. Mass spectrum of BHMFD.

**
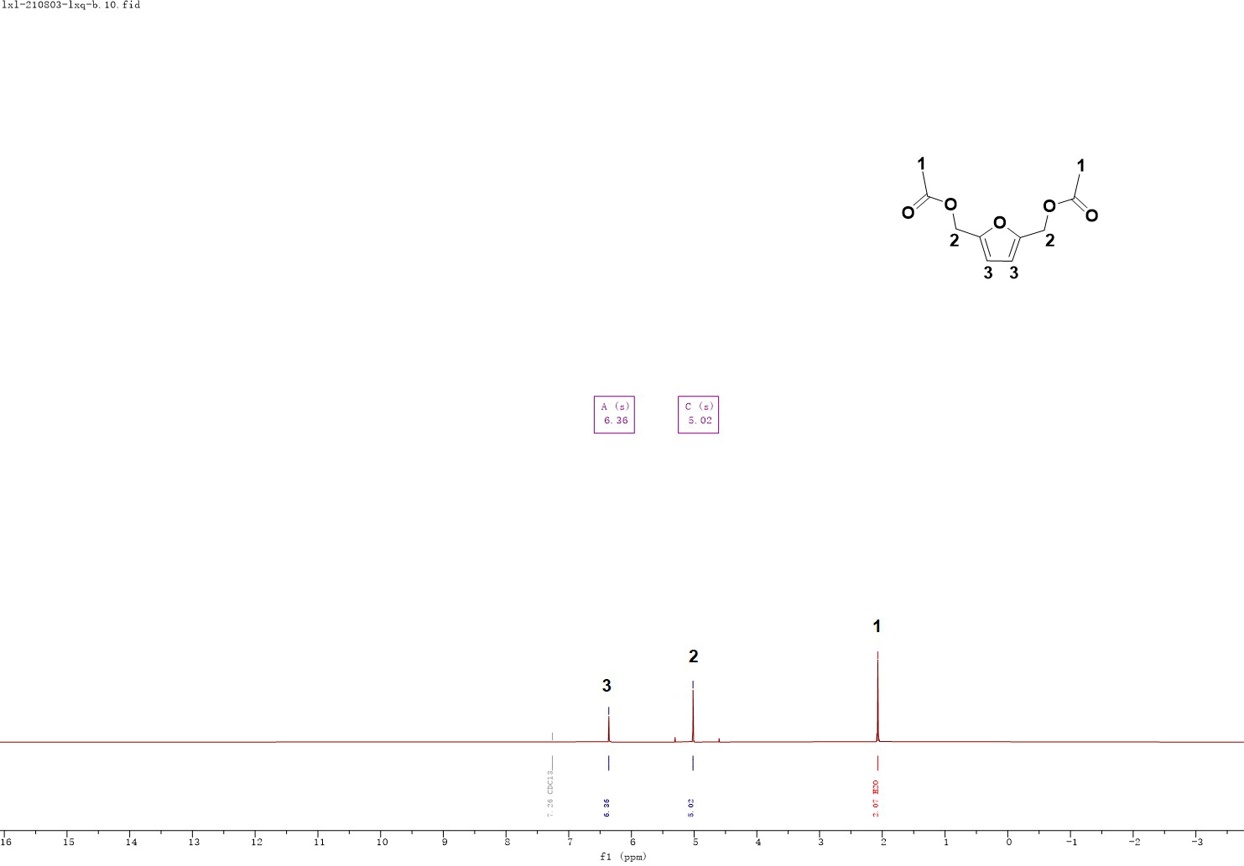
**

Figure S7. NMR spectrum of BHMFD.


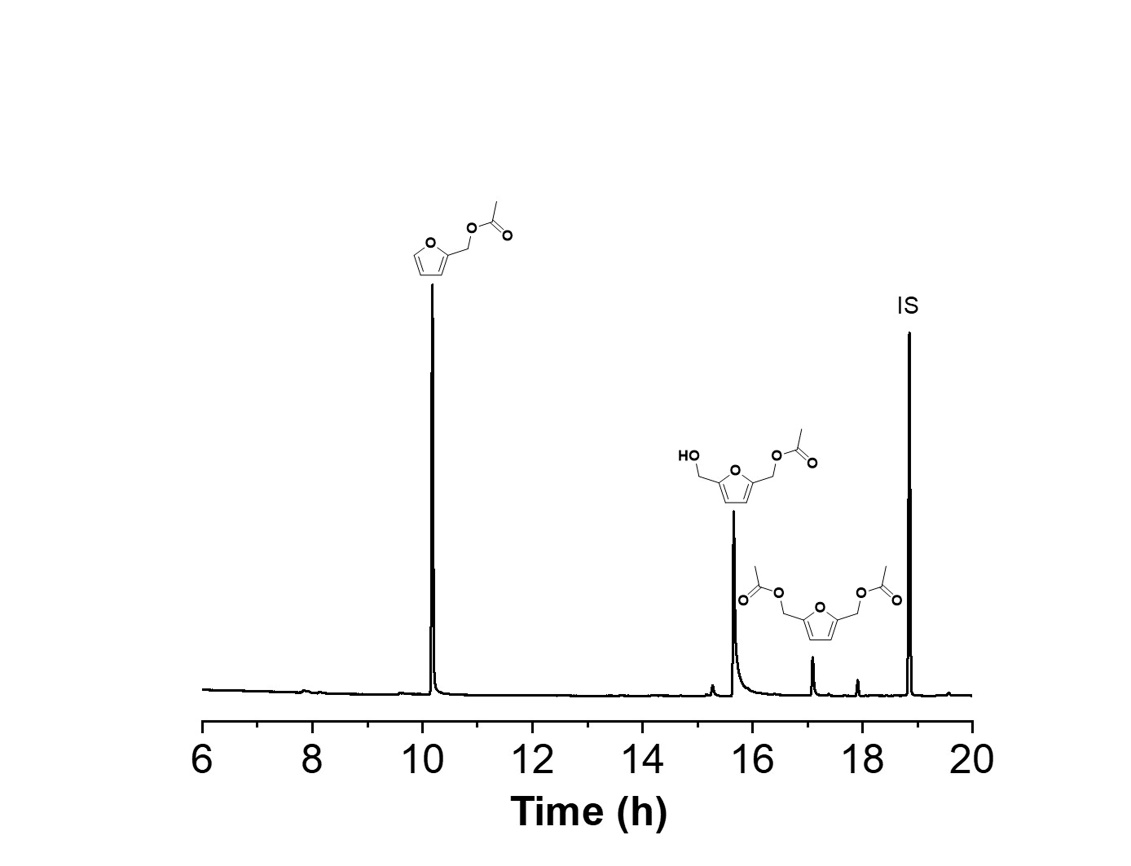


Figure S8. Gas chromatograms of BHMFM and BHMFD. Hydroxylmethylation reaction conditions: 0.4 mmol FA, 1.7 mmol paraformaldehyde and 35.0 mmol CH_3_COOH were reacted at 100^ο^C for 2h. n-hexadecane was used as an internal standard (IS).


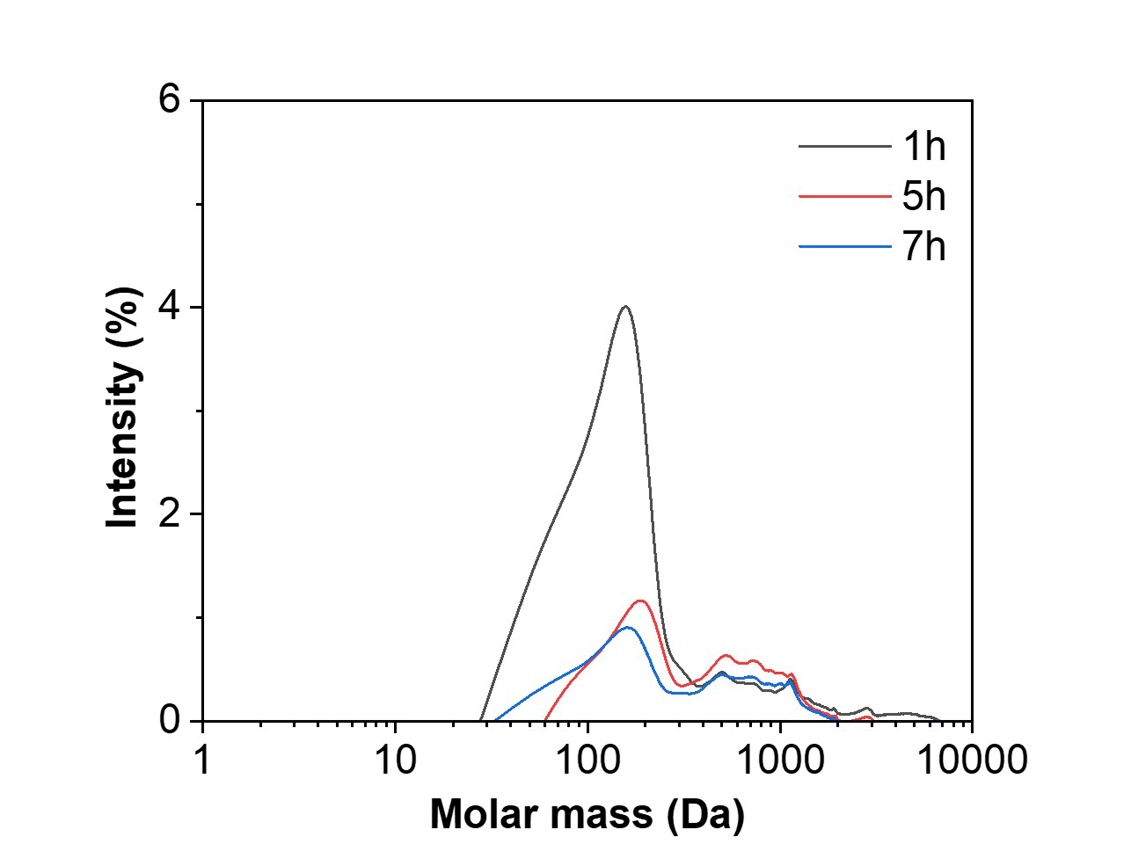


Figure S9. GPC of sample that 0.4 mmol EMF, 1 mL formaldehyde (37 wt% aqueous solution), 0.1 mmol HCl (10 µL of 36 wt% HCl aqueous solution), and 1 mL 1,4-dioxane were reacted at 50^ο^C for 1h, 5h and 7h.

**Table S1. Effects of acid and aldehyde on the hydroxymethylation of EMF.**

| Entry | Acid | Aldehyde | Solvent | Hydroxymethylation products | Carbonization | Reaction liquor |
| --- | --- | --- | --- | --- | --- | --- |
| 1^a^ | HCl  (0.30 wt%) | Formaldehyde (21.73 wt%) | 1,4-dioxane | Detected | Not serious | Yellow |
| 2^b^ | HCl  (0.30 wt%) | Formaldehyde (21.73 wt%) | 1,4-dioxane | Detected | Not serious | Deep yellow |
| 3^a^ | HCl  (0.61 wt%) | Formaldehyde (21.67 wt%) | 1,4-diosane | Detected | Not serious | Brown |
| 4^a^ | HCl  (0.30 wt%) | Formaldehyde (21.26 wt%) and paraformaldehede (4.93 wt%) | 1,4-dioxane | Detected | Not serious | Yellow |
| 5^a^ | HCl  (0.03 wt%) | Formaldehyde (21.79 wt%) | 1,4-dioxane | Not Detected | Not serious | Light yellow |
| 6^c^ | CH_3_COOH (92.54 wt%) | Paraformaldehyde (3.11 wt%) | CH_3_COOH | Detected | Not serious | Yellow |
| 7^d^ | HCOOH  (87.42 wt%) | Formaldehyde (10.81 wt%) | HCOOH | Not Detected | Yes | Black |
| 8^d^ | HCOOH  (46.85 wt%) | Formaldehyde (11.58 wt%) | 1,4-dioxane | Not Detected | Yes | Black |
| 9^a^ | H_2_SO_4_  (1.29 wt%) | Formaldehyde (21.06 wt%) | 1,4-dioxane | Not Detected | Not serious | Light yellow |
| 10^d^ | CF_3_SO_3_H (1.47 wt%) | Paraformaldehyde (4.35 wt%) | 1,4-dioxane | Not Detected | Yes | Black |
| 11^a^ | CF_3_SO_3_H (0.15 wt%) | Paraformaldehyde (4.41 wt%) | 1,4-dioxane | Not Detected | Yes | Black |

Other reaction conditions: a: 50°C; b: 60°C; c: 100°C; d: room temperature.

**Table S2. Effeects of acid and aldehyde on the hydroxymethylations of FA .**

| Entry | Acid | Aldehyde | Solvent | Hydroxymethylation products | Carbonization | Reaction liquor |
| --- | --- | --- | --- | --- | --- | --- |
| 1^a^ | CH_3_COOH (95.20 wt%) | Paraformaldehyde (2.27 wt%) | CH_3_COOH | Detected | Not serious | Golden yellow |
| 2^b^ | CH_3_COOH (4.28 wt%) | Formaldehyde (2.39 wt%) | 1,4-dioxane | Not Detected | Not serious | Yellow |
| 3^a^ | H_2_SO_4_  (7.09 wt%) | Formaldehyde (2.39 wt%) | 1,4-dioxane | Not Detected | Not serious | Black |
| 4^d^ | H_2_SO_4_  (0.14 wt%) | Paraformaldehyde (15.15wt%) | None | Not Detected | Yes | Black |
| 5^a^ | H_2_SO_4_  (0.09 wt%) | Paraformaldehyde (3.63 wt%) | Ethyl acetate | Not Detected | Not serious | Light yellow |
| 6^a^ | H_2_SO_4_  (0.08 wt%) | Paraformaldehyde (2.27 wt%) | CH_3_COOH | Not Detected | Yes | Black |
| 7^e^ | HCOOH (8.47 wt%) | Paraformaldehyde (13.89 wt%) | None | Not Detected | Not serious | Orange |
| 8^f^ | HCOOH (90.65 wt%) | Paraformaldehyde (5.20 wt%) | None | Not Detected | Not serious | Black |
| 9^d^ | CF_3_SO_3_H (0.08 wt%) | Paraformaldehyde (2.30 wt%) | 1,4-dioxane | Not Detected | Yes | Black |
| 10^e^ | CF_3_SO_3_H (0.77 wt%) | Paraformaldehyde (2.28 wt%) | 1,4-dioxane | Not Detected | Yes | Black |
| 11^a^ | HCl  (0.30 wt%) | Fomaldehyde  (21.63 wt%) | 1,4-dioxane | Not Detected | Not serious | Yellow |

Other reaction conditions: a: 50°C; b: 60°C; c: 100°C; d: room temperature; e: 80°C; f: 30°C;

**Table S3. Elemental analysis results.**

| Samples | C (wt %) | H (wt %) | O (wt %) |
| --- | --- | --- | --- |
| EMF-derived oily products before HDO | 55.5 | 5.6 | 38.9 |
| EMF-derived oily products after HDO | 83.7 | 10.4 | 5.9 |
| FA-derived oily products before HDO | 51.8 | 6.2 | 42.0 |
| FA-derived oily products after HDO | 81.0 | 9.3 | 9.7 |
